# Statistical methods for assessing the impact of the Covid-19 pandemic on health services use: Paediatric primary care and mental health access.

**DOI:** 10.23889/ijpds.v7i3.2075

**Published:** 2022-08-25

**Authors:** Therese Stukel, Astrid Guttmann, Natasha Saunders, Longdi Fu

**Affiliations:** 1 ICES; 2 University of Toronto

## Objectives

There were large disruptions to health care services after the onset of the COVID pandemic. We compared outpatient primary care and mental health care physician visits before and during the COVID-19 pandemic in Ontario, Canada.

## Approach

Population-based study of pediatric primary care and mental health visit rates after vs. before Covid restrictions. We used Poisson GEE regression to model 3-year pre-Covid trends and forecast expected trends after restrictions. The model included age, sex, a secular trend, and pre-Covid month indicators. Expected visit rates and 95% CIs post-restrictions were estimated by applying the linear combination of pre-Covid regression coefficients to the post-pandemic data and exponentiating. Relative changes in post Covid visit rates were expressed as an adjusted rate ratio of observed to expected rates by exponentiating the difference of observed and expected post-pandemic log rates and CIs.

## Results

In a population of 2.5 million children, primary care visit rates declined by 20% of expected (adjusted rate ratio [aRR], 0.80; 95% CI, 0.77–0.82). The largest monthly decrease occurred in April 2020. Virtual visits accounted for 53% of overall visits. Although visit rates increased slowly after April 2020, they did not return to pre-restriction levels by November 2020. Mental health visit rates declined rapidly to below expected in April 2020 (aRR, 0.81; 95% CI, 0.79-0.82) followed by an increase to 7% above expected (aRR, 1.07; 95% CI, 1.04-1.09) by July 2020 and sustained at 10-15% above expected to February 2021. Adolescent females had the greatest overall increase in mental health visit rates relative to expected (aRR, 1.26; 95% CI, 1.25-1.28).

## Conclusion

An interdisciplinary approach has been essential for the development and approvals of this project. Bringing together experts from diverse disciplines with different perspectives to use a novel approach enables us to better address the large and important issue of childhood social disparities and their enduring impact on life course health.

